# Performance enhancement of a wind driven PMSG using an artificial neural network based nonlinear backstepping controller

**DOI:** 10.1371/journal.pone.0331976

**Published:** 2025-09-10

**Authors:** Abdelfattah Dani, Zineb Mekrini, Mhamed El Mrabet, Mohammed Boulaala, Hamid Chojaa, Ameena Saad Al-Sumaiti, Mahmoud A. Mossa

**Affiliations:** 1 Industrial Systems Engineering and Energy Conversion Team, FSTT, Abdelmalek Essaadi University, Tetouan, Morocco; 2 Higher School of Technology, Industrial Technologies and Services Laboratory, Sidi Mohamed Ben Abdellah University, Fez, Morocco; 3 Smart OR Lab, Advanced power and energy center, Department of electrical and computer engineering, Khalifa University, Abu Dhabi, United Arab Emirates; 4 Electrical Engineering Department, Faculty of Engineering, Minia University, Minia, Egypt; King Fahd University of Petroleum & Minerals, SAUDI ARABIA

## Abstract

With the increasing demand for wind energy in the electric power generation industry, optimizing robust and efficient control strategies is essential for a wind energy conversion system (WECS). In this regard, this study proposes a novel hybrid control strategy for wind power systems directly coupled to a permanent-magnet synchronous generator (PMSG). The contribution of this work is to propose a control strategy design based on a combination of the nonlinear Backstepping approach for system stabilization according to Lyapunov theory and the application of artificial neural network to maximize energy harvesting regardless of wind speed fluctuations. The hybrid control strategy, which is highly efficient in reducing current/torque ripples, as well as the THD ratio of PMSG currents, was applied to achieve good system performance. The overall system is implemented in MATLAB/Simulink to verify the effectiveness of the proposed control technique under varying wind conditions. Analysis of the simulation results for the proposed control versus field-oriented control (FOC) shows that the proposed control strategy exhibits less ripples in the electromagnetic torque, with the ripple ratio decreasing significantly from 32.95% to 19.43%. In addition, the THDs of the stator current decreased from 20.87% to 14.88%, proving the reliability and efficiency of the proposed control strategy compared to FOC. Meanwhile, these results are valuable for the application of the proposed control strategy in a real WECS system.

## 1. Introduction

In pursuit of sustainable energy solutions, wind turbine (WT) technologies have emerged as a favorite, offering a promising way for clean and renewable power generation. Their scalability and adaptability make them integral to the transition towards sustainable development. From small-scale applications in rural communities to vast offshore and onshore wind farms powering urban centers, wind turbines play a pivotal role in global efforts to reduce carbon emissions and combat climate change [1]. In addition to the environmental benefits, wind power conversion offers economic benefits, including job creation in wind energy manufacturing [2]. Moreover, by harnessing the power of nature’s source, wind turbine technologies exemplify the synergy between innovation and environmental protection, driving us toward a more sustainable future. Globally, installed wind power capacity has expanded significantly over the years, reflecting the rapid increase of this renewable energy source. According to data from the Global Wind Energy Council in a 2024 report, total installed wind power capacity exceeded 1 TW by the end of 2023 [3]. This capacity continues to grow rapidly as new wind farms are developed around the world. Countries such as China, the USA, Brazil, and Germany are among the main contributors to this development. Morocco is one of the nation’s looking to harness the potential of solar and wind power to meet their energy needs while reducing air pollution [4]. On the other hand, maximizing the power production of WT is crucial for enhancing their efficiency and economic capability. In order to achieve this goal, the WECS is managed through three control stages. First, applying maximum power point tracking (MPPT) control, in which the objective is to maximize the extraction of the power that is available in the wind when the wind speed remains within a specific range [5]. Perturb and observe (P&O) algorithm [6], optimal torque control (OTC) [7], and tip speed ratio (TSR) control [8], are widely used, as MPPT control, due to their simplicity and reliability in maintaining desired turbine speed and power output. Next, applying machine-side converter (MSC) control, in which the generator is controlled to rotate within the optimal mechanical speed to generate the optimal electromagnetic torque [9]. Then, applying grid-side converter (GSC) control, in which the primary control objective is to regulate the active and reactive powers to the desired power factor according to grid needs [10]. In the literature, various studies have been done on WECS control strategies [11–13]. These strategies can be broadly categorized into traditional methods and advanced techniques. Generally, classical control strategies include the conventional field-oriented control (FOC) and voltage-oriented control (VOC) based on proportional-integral (PI) regulators [14]. Through the FOC, the electromagnetic torque and flux are controlled by decoupling the stator current into two separate components in the d-q reference frame [15]. The VOC, which is used to inject active power to the electrical grid at the unity power factor (UPF), is also used to maintain the DC-link voltage at its reference value [16]. In addition, the conventional direct torque control (DTC) and direct power control (DPC) based on hysteresis regulators are widely suggested in several research studies for WECS [17,18]. DTC and DPC simplify the control scheme; there is no need for park transformation, the tuning of PI gains, or the pulse width modulation (PWM). DTC directly controls the stator flux and electromagnetic torque through the selection of optimal voltage vectors [19]. DPC directly controls the active and reactive powers according to the optimal voltage vector [20]. The mentioned classical controls usually suffer from slow response, high torque/current ripples, and are sensitive to parameter variation as well as changes in torque and generator speed under variable wind conditions. In order to minimize the impact of these issues, several advanced control strategies have been proposed in the literature. Among them, there are nonlinear techniques based on feedback linearization control (FBLC), H∞ control, sliding mode control (SMC), and backstepping control (BSC) which are emerging as powerful tools for managing the nonlinearities and uncertainties inherent in wind energy conversion systems [21–24]. The feedback linearization approach is based on converting the dynamics of a nonlinear system into a linear system, which simplifies controller design to maintain the system stability [25]. However, the FBLC needs accurate knowledge of the parameters required to model the system [26], which are often unavailable in a real-world application such as WECS. H∞ control is another nonlinear control technique applied in WECS technologies [27]. It is a robust control that describes the control objective as an optimization problem and returns the solution. Additionally, it can be directly applied to multivariate systems with coupled states [28]. However, a precise WECS model and a deep understanding of the control structure are necessary for H-infinity control. SMC is a robust nonlinear control strategy introduced by Vadim. I. Utkin in the 1970s [29]. This control strategy is designed to efficiently handle nonlinear systems by guiding the system states to a predefined sliding surface. SMC ensures insensitivity to disturbances and changes in system parameters. However, chattering phenomenon and high-frequency oscillations caused by the discontinuous nature of the control signal are the main drawbacks of SMC [30,31]. These limitations directly affect the control efficiency of global WECS, which reflects the increased harmonics in currents and torque ripples under variable wind conditions [32]. Similarly, another nonlinear technique named the Backstepping technique was developed by Kanellakopoulos and Kokotovic in 1990 [33]. The principle of BSC control is to transform a complex nonlinear system into a cascading succession of simple subsystems. At the same time, it provides virtual control of each subsequent subsystem. In the design process, intermediate stabilizing control laws are obtained from the well-known Lyapunov function to guarantee the stability of the resulting control laws, enabling the system to converge towards its stable state [34]. However, this technique suffers from computational complexity known as ‘’ explosion of terms’ [35]. Recently, BSC technique has been increasingly proposed in literature related to wind energy conversion systems [36–39]. Moreover, linear quadratic gaussian (LQG) control, linear quadratic regulator (LQR), and model predictive control (MPC) are among the optimal control techniques that are widely applied in the WECS [40–43]. MPC is particularly adopted in various research studies to predict the behavior of a control system in response to changes in its parameters [42]. Despite its advantages, including constraint handling, fast response, low maintenance cost, and optimal energy extraction, MPC has some limitations and potential challenges, such as computational complexity and sampling times that may be too long for highly dynamic systems [44,45]. Currently, soft computing techniques are the most popular methods developed to improve the performance of wind energy extraction systems. These artificial intelligence (AI) techniques include fuzzy logic control (FLC), artificial neural networks (ANN), and adaptive neural network-based fuzzy inference systems (ANFIS). The FLC structure comprises three processes: fuzzification, inference, and defuzzification. FLC depends on the designer’s ability to identify the precise membership function for the appropriate error. Despite its simplicity and adaptability, FLC suffers from a memory allocation problem and a lack of stability guarantees during sudden changes in wind speed [46]. The structure of an ANN controller comprises an input layer, a hidden layer, and an output layer. This AI-based controller is increasingly explored in the literature for controlling WECS [47]. ANN is known for its efficiency and rapid response capability, but its main limitations are its complex architecture and the training time required. Readers interested in the history of artificial neural networks and their applications can refer to reference [48]. ANFIS is another intelligent controller based on the combination of ANN and FLC in managing complex control design. Fuzzy rules can be used to dynamically adjust the gain to improve the efficiency of handling system uncertainties [49].

Recently, hybrid control strategies have become the most popular methods developed to reduce the limitations and overcome the control issues of the above-mentioned techniques. That is achieved by addressing the weaknesses of each technique through the other. In the literature, many research papers propose hybrid control techniques based on a nonlinear controller and AI to enhance the WECS efficiency and performance. In this context, the following studies refer to the combination of BSC as a nonlinear controller and AI-based techniques for wind energy extraction. In [50], the authors developed a fuzzy control algorithm that adapts to the fractional order of DFIG to mitigate the impact of external disturbances and model uncertainty on the power transferred to the grid. This method is based on combining BSC technique with two fuzzy Takagi–Sugeno systems in fractional order. The main advantages of this method are fast response, overall system stability, and excellent tracking performance. But the ability to handle appropriate mathematical tools is essential for system modeling and control design. In [51], a self-tuning BSC system with FLC parameters is developed to enable adaptive adjustment of generator speed control parameters in order to improve the performance and efficiency of the BSC strategy for a dual grid-connected WECS based on two 5-phase PMSG-based WECS systems. This proposed work reveals several improvements such as: THD percentage, dynamic response power, overshoot, steady-state error, as well as current and power ripples. However, it is a complex technique to implement. Another hybrid intelligent BSC suggested in the literature lies in the use of ANN and BSC [52]. The authors used an RBFNN-based disturbance observer to estimate unknown disturbances of the system. This approach combines RBFNN with fractional-order backstepping SMC to control the generator speed in a DFIG-based WECS. Despite the complexity of the modeling and implementation structures of this method, it offers several advantages, such as high efficiency, robustness against chattering, and the ability to handle unknown disturbances and parameter uncertainties. In [53], a new adaptive hybrid control system is developed for the PMSG-based WECS to extract maximum energy. The main idea of this method is to approximate the optimal mechanical torque value using a neural network identifier. Next, the estimated wind speed is calculated using the approximate torque. Additionally, a nonlinear BSC is derived to adjust the rotor speed to its optimal value, thereby achieving maximum power point tracking and ensuring system stability. However, this method reveals two difficult points: the need to estimate or measure the mechanical torque, as well as to operate the system at the optimal mechanical torque/speed point. In [54] a hybrid controller based on backstepping and an adaptive neural network is proposed to extract maximum wind energy through the DFIG-based WECS. Principle of this method: The ANN is used to estimate the value of various uncertainty parameters in the system, and the weight adaptation algorithms are based on the integration law. The final backstepping control law is based on the ANN estimation and Lyapunov stability theory. This robust control strategy ensures good performance in terms of response time and reference tracking, but it is complex to implement. In [55], an adaptive BSC control is developed and integrated with neural networks for a PMSG-based WECS system to control rotor speed and generated mechanical power. Two ANN compensators are created to compensate for uncertainty in the generator current loops. This method effectively addresses uncertainty and ensures system stability, but it suffers from both discrete system design and implementation complexity. In addition, an ANN-based speed optimizer and a BSC controller are combined to establish a hybrid control strategy in [56]. The objective of this suggested control is to extract the maximum power from the available wind energy using a PMSG direct-drive wind turbine. This hybrid technique is effective in achieving the desired control objectives, such as low THD. However, this strategy cannot eliminate the steady-state error, especially for the stator current. A recent study presented a hybrid control strategy that combines ANN-based MPPT and BSC to improve the performance of PMSG-based WECS [57]. The objective of this study is to utilize an ANN controller instead of a PI controller to regulate the mechanical speed. Then, the BSC is applied to the MSC converter, which eliminates overshoot and enhances the system’s response speed and stability. However, a large ripple appears in the stator current.

Hybrid control strategies based on nonlinear BSC with intelligent techniques, such as ANN, for PMSG-based WECS have not been sufficiently reported in the literature. The backstepping approach ensures Lyapunov stability through construction, while ANN is a universal approach that can model highly complex systems. Therefore, combining the advantages of ANN and BSC can ensure stability and guarantee high performance of the direct-drive PMSG-based WECS under variable wind conditions. Motivating by the aforementioned gaps and to contribute to a useful background for future research development in the field of BSC intelligent hybrid control for WECS, this paper proposes a hybrid ANN backstepping control to minimize the current/torque ripple of the PMSG and achieve good system performance, thereby maximizing energy harvesting regardless of wind speed variations. The proposed technique is based on a nonlinear backstepping approach combined with an ANN-based MPPT controller (ANN-BSC). The focus of this study lies on the modelling and control of PMSG-based WECS using an ANN controller for the MPPT part and a backstepping control for the MSC. Moreover, a conventional control strategy based on the OTC controller for the MPPT part and the MSC relies on a field-oriented control, referred to as OTC-FOC, which is highlighted and modelled to be compared with the suggested control strategy. The high efficiency, high performance, and superiority of the proposed technique versus OTC-FOC are verified by simulation results using the Simulink/MATLAB software environment.

The main elements contributed by this article can be briefly summarized as follows:

- Present a nonlinear mathematical model of PMSG and the wind turbine.

Introduce and explain the operational principles of the Backstepping control theory.

- Develop and design an innovative, robust nonlinear hybrid control strategy that combines the ANN and backstepping control.
- Overall enhancement in the performance of the entire system:
  - Maximum power extraction through the MPPT control.
  - Improving the rise time, transient time, overshoot, undershoot, current/ torque ripples, as well as the current THD percentage.
  - Validate the superiority of the proposed control strategy through MATLAB/Simulink software using a variable wind profile.

This research paper is organized as follows: Section 2 presents the principle of WECS and the mathematical model of a direct-drive horizontal-axis wind turbine based on PMSG. Section 3 focuses on control strategies applied to the system, in which the principle and design of the suggested hybrid control strategy are presented. Furthermore, the results analysis, discussion, and comparison are given in Section 4. Finally, Section 5 provides perspectives and a brief conclusion of this paper.

## 2. Direct drive WECS modeling

**Fig 1:**
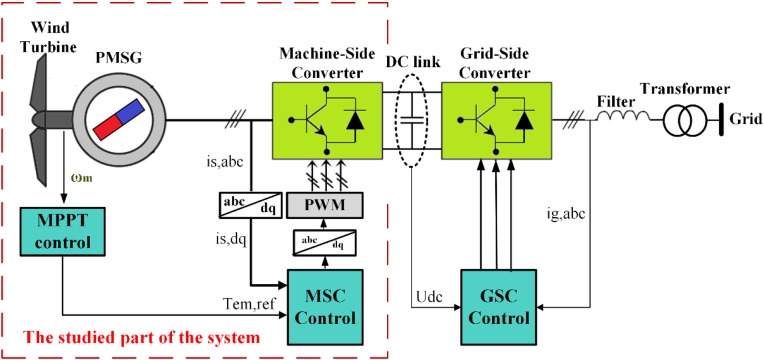
Schematic representation of WECS.

The structure of the PMSG-based direct-drive WECS is shown schematically in Fig 1, where the variable-speed wind turbine is directly coupled to a PMSG generator to generate electrical power from the wind energy to the grid via electrical power converters, filter and a transformer [58].

### 2.1 WT model

The aerodynamic power $P_{aer}$, also called mechanical power, is generated when turbine blades rotate due to the force of the wind. It plays a crucial role in converting wind energy into usable electrical power. This power can be expressed using the following formula [59]:


$$P_{aer}=\frac{1}{2}\rho \pi R^{2}C_{p}(\lambda ,\beta )\upsilon^{3}$$ (1)

This power depends on various factors like wind speed υ, blade design (R radius of WT blades), air density ρ and power coefficient $C_{p}(\lambda ,\beta )$. So that $C_{p}$ varies according to λ the TSR and the blade pitch angle denotes β.


$$\lambda =\frac{R.\omega_{m}}{\upsilon }$$ (2)

The mechanical torque $T_{m}$ of the WT is clearly depend of the wind speed, as indicate in the following formula:


$$T_{m}=\frac{P_{aer}}{\omega_{m}}=\frac{\rho \pi R^{2}C_{p}(\lambda ,\beta )}{{2\omega }_{m}}\upsilon^{3}$$ (3)

With $\omega_{m}$ denote the mechanical angular speed.

The power coefficient Cp describes the theoretical limit of Betz, i.e., the maximum power extracted from the wind is 59.25%. Cp has several approximations in the literature, depending on the dynamics of the WT [60]. Among its mathematical models commonly used in the literature is the following expression:


$$\left\{ \;\;\;\begin{array}{c} C_{p}(\lambda ,\beta )=h_{1}\left(\frac{h_{2}}{A}-h_{3}\beta -h_{4}\right)e^{-\frac{h_{5}}{A}}+h_{6}\lambda \\ \frac{1}{A}=\frac{1}{\lambda +0.08\beta }-\frac{0.035}{\beta^{3}+1} \end{array}\right.$$ (4)

Where the coefficients $h_{1}=\text{0}.\text{5176}$; $h_{\text{2}}=\text{116}$;$h_{\text{3}}=\text{0}.\text{4}$; $h_{\text{4}}=\text{5}$; $h_{\text{5}}=\text{21}$ and $h_{\text{6}}=\text{0}.\text{0068}$.

**Fig 2:**
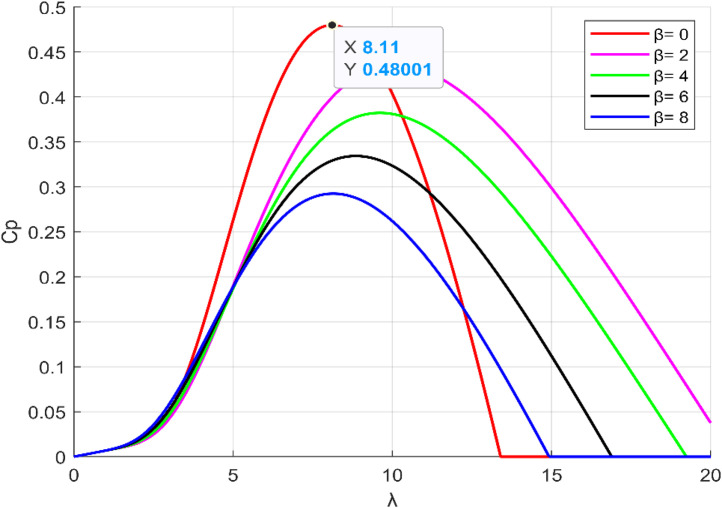
The ${𝐂}_{p}(\lambda ,\beta )$ evolution.

According to the Fig 2 which presents the evolution of the $C_{p}$ as a function of λ and β. It is possible to observe that for each $C_{p}$ curve, an optimal TSR corresponds to a maximum power coefficient. From this figure, it can be noted that for an optimal $\lambda_{opt}=\text{8},\text{1}$ and β = 0^◦^ the power coefficient is at its maximum value $C_{p,max}=\text{0},\text{48}$.

### 2.2 PMSG model

The two main components of the permanent-magnet synchronous machine (PMSM) are the wound stator and the permanent-magnet rotor, which is usually located inside the stator. The operating principle of this type of machine is the interaction between the constant magnetic field of the rotor and the rotating magnetic field of the stator. Electromagnetic torque is produced by this interaction and reaches its maximum when the magnetic vectors of the stator and rotor are perpendicular to each other [61].

In general, some assumptions are made to simplify the mathematic model of PMSM, such as ignoring magnetic saturation or simplifying the effects of harmonic distortion. Furthermore, it is often assumed that the stator windings are uniformly distributed in three phases [62].

The mathematical model of a PMSG in the (d,q) reference frame can be represented as follows [63]:


$$\left\{ \;\;\;\begin{array}{c} v_{sd}=R_{s}i_{sd}+\frac{d}{dt}(\psi_{d})-\omega_{r}\psi_{q}\mathrm{\backslash vspace}2mm\\ v_{sq}=R_{s}i_{sq}+\frac{d}{dt}(\psi_{q})+\omega_{r}\psi_{d} \end{array}\right.$$ (5)

$$\left\{ \;\;\;\begin{array}{c} \psi_{d}=L_{sd}i_{sd}+\varphi_{r}\\ \psi_{q}=L_{sq}i_{sq} \end{array}\right.$$ (6)

$$\left\{ \;\;\;\begin{array}{c} v_{sd}=R_{s}i_{sd}+L_{sd}\frac{di_{sd}}{dt}-\omega_{r}L_{sq}i_{sq}\mathrm{\backslash vspace}2mm\\ v_{sq}=R_{s}i_{sq}+L_{sq}\frac{di_{sq}}{dt}+\omega_{r}L_{sd}i_{sd}+\omega_{r}\varphi_{r} \end{array}\right.$$ (7)

$$T_{em}=\text{1},\text{5}p\left(\left(L_{sd}-L_{sq}\right)i_{sd}i_{sq}+\varphi_{r}i_{sq}\right)$$ (8)

$$J\frac{d\omega_{m}}{dt}+f_{c}\omega_{m}=T_{m}-T_{em}$$ (9)

$$P_{s}=\frac{3}{2}\left(v_{sd}i_{sd}+v_{sq}i_{sq}\right)$$ (10)

$$Q_{s}=\frac{3}{2}\left(v_{sq}i_{sd}-v_{sd}i_{sq}\right)$$ (11)

Where $\left(v_{sd},v_{sq}\right)$, $\left(i_{sd},i_{sq}\right)$,$\left({\psi_{d},\psi }_{q}\right)$, $\left(L_{sd},L_{sq}\right)$ are (d,q) axis components of voltage, current, generator flux and stator inductance respectively; $R_{s}$ refers to the stator resistance; $T_{em}$ is the electromagnetic torque; p is the number of PMSG pole pairs; J refers to the moment inertia of the (WT-PMSG) system; $\omega_{r}=p\omega_{m}$ is the electrical rotation speed; $\varphi_{r}$ is the PMSG rotor flux and $f_{c}$ is the coefficient of friction. $P_{s}$ and $Q_{s}$ are the stator active and reactive powers, respectively.

## 3. Design system control strategies

### 3.1 MPPT control principle

The main objective of MPPT control strategies is to optimize the power coefficient to maximize wind turbine efficiency with respect to the wind behavior, which can be considered stochastic [64].

Applying Optimal Torque Control which is one of the MPPT control techniques used in WECS. Even without directly measuring wind speed, this technique regulates generator torque while adjusting turbine speed to predefined values [65]. In addition, it is essential to set the TSR and power coefficient to their respective optimum values in order to estimate the wind speed value for maximum power extraction. With the following equations, the instantaneous estimate of the wind speed $\hat{\upsilon }$ can be represented using the estimated mechanical torque $T_{m}^{est}$. Thus, it can be calculate the electromagnetic torque $T_{em}^{ref}$ that enables the system to perform around its optimal speed [66]:


$$\hat{\upsilon }=\frac{\text{R}.\omega_{\text{m}}}{\lambda_{\text{opt}}}$$ (12)

$$T_{m}^{est}=\frac{1}{2}\frac{\rho \pi R^{2}C_{p,max}}{\omega_{m}}{\hat{\upsilon }}^{3}$$ (13)

$${\text{T}}_{\text{em}}^{\text{ref}}=\frac{\text{1}}{\text{2}}\frac{\rho \pi {\text{R}}^{\text{5}}{\text{C}}_{\text{p},\text{max}}}{\lambda_{\text{opt}}^{\text{3}}}{\omega_{\text{m}}}^{\text{2}}$$ (14)

The tip speed ratio control strategy stands out as a widely utilized MPPT technique for managing wind turbines operating at variable speeds. Initially, wind speed is measured using an anemometer. Subsequently, by setting the TSR to it optimal value, the optimal rotor speed $\omega_{m}^{ref}$ is generated [67]. In addition, setting the mechanical speed error as the input of a controller produced the desired electromagnetic torque to meet the system’s requirements.

### 3.2 OTC-FOC strategy

This strategy focuses on controlling PMSG-based WECS using OTC controller for MPPT part and the MSC relies on field-oriented control (FOC), which is based on a proportional integral controller (PI), to control the electromagnetic torque via stator currents, hence regulating the mechanical speed of the PMSG. This method eliminates the coupling between the direct and quadrature axis components that make the PMSG’s behavior similar to that of a DC machine. The main idea is to use the direct component of the stator currents to regulate the magnetic flux. While the electromagnetic torque is adjusted via the quadrature component [68]. Generally, the direct axis current component must be set to zero to validate the implementation of this vector control strategy [69]. In addition, equation (15) reformulated and expressed the electromagnetic torque. This mathematical expression emphasizes that the electromagnetic torque is affected only by the quadratic component of the stator currents.


$$T_{em}=\frac{\text{3}p\varphi_{r}}{\text{2}}i_{sq}$$ (15)

### 3.3 Nonlinear backstepping control

#### a. Principle.

Backstepping is a control strategy that is beneficial for dealing with nonlinear systems. The principle behind the backstepping technique is to make a complex nonlinear system equivalent to simple cascaded subsystems [70]. At the same time, each subsystem provides virtual control to the next. It is based on designing a control law for the transformed system by recursively selecting Lyapunov functions to stabilize the whole system [71].

#### b. Design of BSC.

The BSC strategy for optimizing energy extraction in a PMSG-based WECS retains the same fundamental control principles of the backstepping approach. Hence, to design a nonlinear controller to produce the electromagnetic torque required to ensure maximum power extraction and system stability at the same time, it is necessary to accomplish the following steps:

- Define the error variables and the required trajectories.
- Determine a Lyapunov function to ensure the system’s stability.
- Iteratively design virtual control laws to stabilize each subsystem.

***Step 1: Backstepping speed controller***

The rotational speed tracking error and its derivative can be defined as follow:


$$\varepsilon_{\omega }=\omega_{m}^{ref}-\omega_{m}$$ (16)

$${\dot{\varepsilon }}_{\omega }={\dot{\omega }}_{m}^{ref}-\frac{\text{1}}{J}\left(T_{m}-T_{em}-f_{c}\omega_{m}\right)$$ (17)

The following equation presents the expression of the Lyapunov candidate function:


$$V_{\text{1}}=\frac{\text{1}}{\text{2}}\varepsilon_{\omega }^{\text{2}}$$ (18)

The time derivative of the Lyapunov candidate function can be expressed as follow:


$${\dot{V}}_{\text{1}}=\left[{\dot{\omega }}_{m}^{ref}-\frac{\text{1}}{J}\left(T_{m}-T_{em}-f_{c}\omega_{m}\right)\right].\varepsilon_{\omega }$$ (19)

$\gamma_{\text{1}}$ should be chosen as positive constant to guarantee the system stability in Lyapunov sense, that mean ${\dot{V}}_{\text{1}}$ is equal to a negative value [72]. By applying the following stabilizing control law (equation 20) to ensure the system’s stability by satisfying the Lyapunov condition:


$$T_{em}^{ref}=T_{m}{-J\dot{\omega }}_{m}^{ref}-f_{c}\omega_{m}-\gamma_{\text{1}}\text{J}\varepsilon_{\omega }$$ (20)

Therefore, this control law is designed to make the Lyapunov function’s derivative negative definite, ensuring asymptotic stability.


$${\dot{V}}_{\text{1}}=-\gamma_{\text{1}}\varepsilon_{\omega }^{\text{2}}\leq \text{0}$$ (21)

***Step 2: Backstepping Current Controller***

Assuming stator inductances are equal $L_{sd}=L_{sq}$ and ${\dot{\omega }}_{m}^{ref}=\text{0}$, the electromagnetic toque can be expressed as follow:


$$T_{em}=\frac{\text{3}p\varphi_{r}}{\text{2}}i_{sq}$$ (22)

Hence, by using equations 20 and 22, inputs of the second subsystem can be defined as follow:


$$\left\{ \;\;\;\begin{array}{c} i_{sd}^{*}=\text{0}\\ i_{sq}^{*}=\frac{\text{2}}{\text{3}p\varphi_{r}}\left[T_{m}-f_{c}\omega_{m}-\gamma_{\text{1}}\text{J}\varepsilon_{\omega }\right] \end{array}\right.$$ (23)

The stator current errors and their time derivatives are represented by the following equations:


$$\left\{ \;\;\;\begin{array}{c} \varepsilon_{d}=i_{sd}^{*}-i_{sd}\\ \varepsilon_{q}=i_{sq}^{*}-i_{sq} \end{array}\right.$$ (24)

$$\left\{ \;\;\;\begin{array}{c} i_{sd}=-\varepsilon_{d}\\ i_{sq}=i_{sq}^{*}-\varepsilon_{q} \end{array}\right.$$ (25)

$${\dot{\varepsilon }}_{\omega }=-\frac{\text{1}}{J}\left(T_{m}-\frac{\text{3}p\varphi_{r}}{\text{2}}i_{sq}-f_{c}\omega_{m}\right)=-\frac{\text{1}}{J}\left(T_{m}-\frac{\text{3}p\varphi_{r}}{\text{2}}\left(i_{sq}^{*}-\varepsilon_{q}\right)-f_{c}\omega_{m}\right)$$ (26)

$${\dot{\varepsilon }}_{\omega }=-\gamma_{\text{1}}\varepsilon_{\omega }-\frac{\text{3}p\varphi_{r}}{\text{2}J}\varepsilon_{q}$$ (27)

$${\dot{\varepsilon }}_{d}=-\frac{\text{d}i_{sd}}{dt}=\frac{\text{1}}{L_{sd}}\left(R_{s}i_{sd}-{p\omega }_{m}L_{sq}i_{sq}-v_{sd}\right)$$ (28)

$${\dot{\varepsilon }}_{q}=\frac{\text{d}i_{sq}^{*}}{dt}-\frac{\text{d}i_{sq}}{dt}=\frac{\text{2}}{\text{3}p\varphi_{r}}\left[{\dot{T}}_{m}-f_{c}{\dot{\omega }}_{m}-\gamma_{\text{1}}\text{J}{\dot{\varepsilon }}_{\omega }\right]+\frac{\text{1}}{L_{sq}}\left(R_{s}i_{sq}+{p\omega }_{m}L_{sd}i_{sd}+{p\omega }_{m}\varphi_{r}-v_{sq}\right)$$ (29)

Replacing ${\dot{\omega }}_{m}$ and ${\dot{\varepsilon }}_{\omega }$ by their expression using equations 9, 22 and 27.


$${\dot{\varepsilon }}_{q}=\frac{\text{2}}{\text{3}p\varphi_{r}J}\left[J{\dot{T}}_{m}+\left(\text{J}\gamma_{\text{1}}-f_{c}\right)\left(T_{m}-\frac{\text{3}p\varphi_{r}}{\text{2}}i_{sq}-f_{c}\omega_{m}\right)\right]+\frac{\text{1}}{L_{sq}}\left(R_{s}i_{sq}+{p\omega }_{m}L_{sd}i_{sd}+{p\omega }_{m}\varphi_{r}-v_{sq}\right)$$ (30)

Control lows designing using the following Lyapunov candidate function:


$$V_{\text{2}}=\frac{\text{1}}{\text{2}}\left(\gamma_{\text{1}}\varepsilon_{\omega }^{\text{2}}+{\gamma_{\text{2}}\varepsilon }_{d}^{\text{2}}+{\gamma_{\text{3}}\varepsilon }_{q}^{\text{2}}\right)$$ (31)

The derivative of Lyapunov function can be expressed as follow:


$${\dot{V}}_{\text{2}}={\dot{\varepsilon }}_{\omega }\varepsilon_{\omega }+{\dot{\varepsilon }}_{d}\varepsilon_{d}+{\dot{\varepsilon }}_{q}\varepsilon_{q}$$ (32)

$$\begin{array}{cc} {\dot{V}}_{\text{2}} & =-\left(\gamma_{\text{1}}\varepsilon_{\omega }^{\text{2}}+{\gamma_{\text{2}}\varepsilon }_{d}^{\text{2}}+{\gamma_{\text{3}}\varepsilon }_{q}^{\text{2}}\right)+\left(\gamma_{\text{1}}\varepsilon_{\omega }^{\text{2}}+{\gamma_{\text{2}}\varepsilon }_{d}^{\text{2}}+{\gamma_{\text{3}}\varepsilon }_{q}^{\text{2}}\right)-\gamma_{\text{1}}\varepsilon_{\omega }^{\text{2}}-\frac{\text{3}p\phi_{r}}{\text{2}J}\varepsilon_{q}\varepsilon_{\omega }+\frac{\varepsilon_{d}}{L_{d}}\left(R_{s}i_{sd}-{p\omega }_{m}L_{sq}i_{sq}-v_{sd}\right)\\ & +\frac{\text{2}\varepsilon_{q}}{\text{3}p\phi_{r}J}\left[J{\dot{T}}_{m}+\left(\gamma_{\text{1}}-f_{c}\right)\left(T_{m}-\frac{\text{3}p\phi_{r}}{\text{2}}i_{sq}-f_{c}\omega_{m}\right)\right]+\frac{\varepsilon_{q}}{L_{sq}}\left(R_{s}i_{sq}+{p\omega }_{m}L_{sd}i_{sd}+{p\omega }_{m}\phi_{r}-v_{sq}\right) \end{array}$$ (33)

${\dot{V}}_{\text{2}}$ must be negative to ensure the system stability.


$${\dot{V}}_{\text{2}}=-\left(\gamma_{\text{1}}\varepsilon_{\omega }^{\text{2}}+{\gamma_{\text{2}}\varepsilon }_{d}^{\text{2}}+{\gamma_{\text{3}}\varepsilon }_{q}^{\text{2}}\right)\leq \text{0}$$ (34)

The following stabilizing control laws lead to satisfaction of the Lyapunov condition, where $\gamma_{j}$ is positive constant with (j=1,2,3):


$$v_{sd}^{*}=R_{s}i_{sd}-{p\omega }_{m}L_{sq}i_{sq}+L_{sd}\gamma_{\text{2}}\varepsilon_{d}$$ (35)

$$v_{sq}^{*}=-\frac{\text{3}pL_{sq}\phi_{r}}{\text{2}J}\varepsilon_{\omega }+\frac{\text{2}L_{sq}}{\text{3}p\phi_{r}}\left[{\dot{T}}_{m}+\left(\gamma_{\text{1}}-\frac{f_{c}}{J}\right)\left(T_{m}-\frac{\text{3}p\phi_{r}}{\text{2}}i_{sq}-f_{c}\omega_{m}\right)\right]+R_{s}i_{sq}+{p\omega }_{m}L_{sd}i_{sd}+{p\omega }_{m}\phi_{r}+L_{sq}\gamma_{\text{3}}\varepsilon_{q}$$ (36)

### 3.4 Hybrid control strategy

#### a. Principles of ANN.

Artificial Neural Networks are powerful nonlinear data approximation techniques that have proven their value in many fields including control systems. ANNs are an architecture that is more or less inspired by how the human brain works, and are mainly based on the concept of a neuron. An ANN consists of interconnected neurons with weighted connections and uses activation functions to produce a nonlinear dynamic [73]. Thus, it allows the network to understand the relationship between input and output signals in complex systems. Two fundamental mathematical operations are carried out to achieve that: summing the weighted inputs and then applying a nonlinear activation function to the result. The mathematical model of a neuron structure can be defined as follows [74].


$$y=a\left(\sum_{r}^{n}w_{r}x_{r}+b\right)$$ (37)

**Fig 3:**
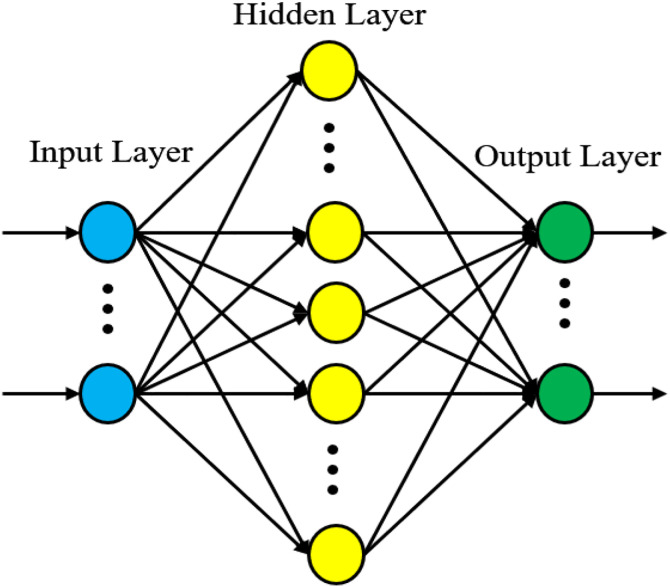
ANN architecture.

The parameters y, a, n, x{$x_{r}$, r = 1, 2,…, n}, $w_{r}$ and b are the neuron output signal, the activation function, the number of neuron inputs, input vector signals of neuron, the weights matrix, and the bias respectively. The most popular ANNs are the multilayer feed-forward neural networks trained by the backpropagation algorithm. Generally, the multilayer is organized into: an input layer, a hidden layer, and an output layer, each layer made up of a several neurons, as depicted in Fig 3.

#### b. Proposed ANN-BSC strategy.

**Fig 4:**
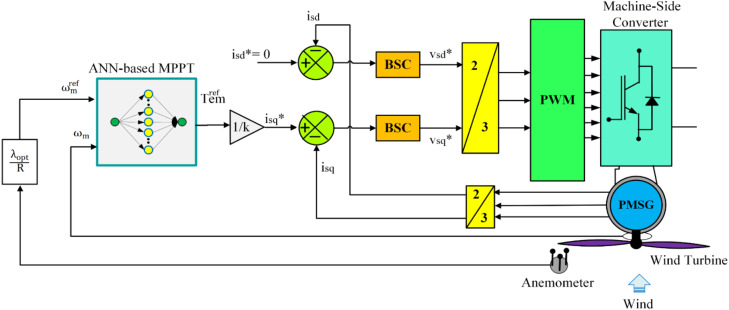
Illustration of BSC control using ANN for WECS.

The basic idea of the proposed control strategy is to design a hybrid control system by replacing the BSC-based MPPT part with an artificial neural network while maintaining the nonlinear BSC control in the MSC part. This aims to minimize the torque and current ripples in the generator, thereby increasing the performance of the WECS. The block diagram of the proposed hybrid nonlinear BSC control, applied to machine-side converter, is shown in Fig 4.

**Fig 5:**
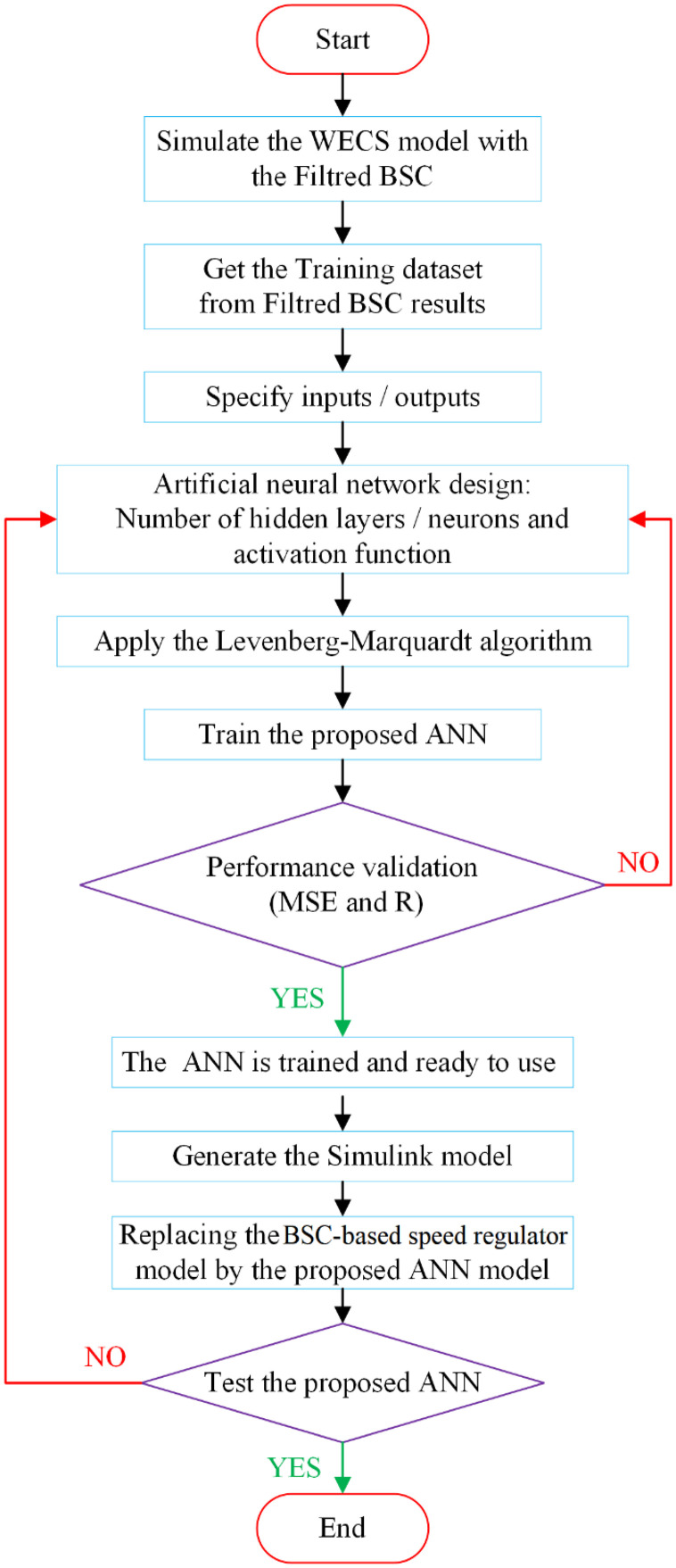
Training process flowchart of ANN-based MPPT controller.

The main steps to design the proposed hybrid control strategy are shown in the flowchart in Fig 5. Before designing the ANN-based MPPT controller, to avoid oversize signal of ${\text{T}}_{\text{em}}^{\text{ref}}$ in the initial phase of control system operation, the reference electromagnetic torque signal (the virtual control signal) is filtered through a first-order low-pass filter of the Simulink block library. The datasets utilized in this instance were obtained from the simulation results of filtered BSC, particularly the input and output values of the first subsystem (system’s speed regulator).

In this study, the dataset was randomly divided into three separate subsets for training, validation, and testing purposes. Furthermore, Table 1 illustrates the detailed configuration of the proposed neural network. In this context, mean squared error (MSE) is used as the primary performance parameter to assess the effectiveness of the created network model. The average squared discrepancies between the ANN’s outputs and the actual target values are measured by MSE. Finding the optimal ANN controller during simulation is achieved by obtaining the lowest MSE, which shows a better fit between the model’s predictions and the target data. The Levenberg-Marquardt backpropagation algorithm is used to iteratively modify the weights throughout the training phase. Through each epoch, this correction is made until the error levels are within a reasonable range. The neural network design is adjusted by changing the number of neurons in the hidden layer or/and the number of hidden layers if the error does not sufficiently converge to zero.

**Table 1:** ANN architecture and configuration.

ANN architecture		Multi-Layer Perceptron Feedforward
Neuron Numbers	Input layer	2
	Hidden layer 1	8
	Hidden layer 2	10
	Hidden layer 3	5
	Output layer	1
Inputs data		$\omega_{\text{m}}^{\text{ref}},\omega_{\text{m}}$
Outputs data		${\text{T}}_{\text{em}}^{\text{ref}}$
Hidden activation		Tansig
Output activation		Purline
Training dataset		70%
Validation dataset		15%
Testing dataset		15%
Training algorithm		Levenberg-Marquardt algorithm (Trainlm)
Number of epochs		300
Learning rate		0.1
Training goal		1e-7
Performance function		MSE: 1.06e-3

## 4. Analysis and discussion of simulation results

**Fig 6:**
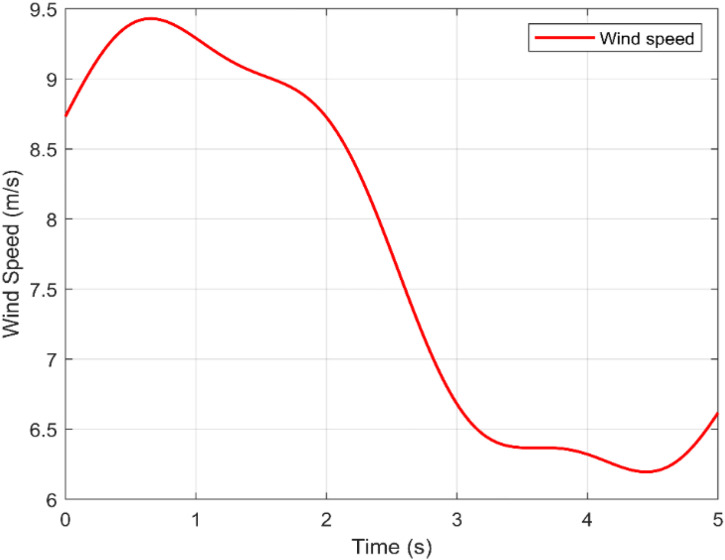
Wind speed profile.

**Fig 7:**
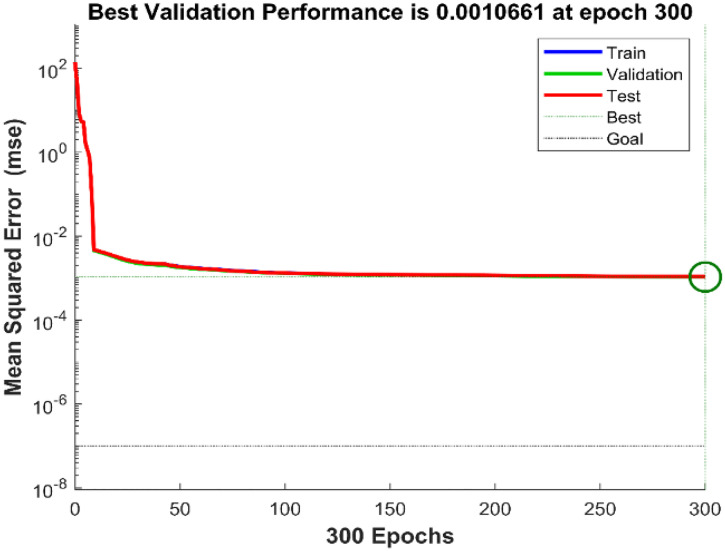
Training performance (MSE).

**Fig 8:**
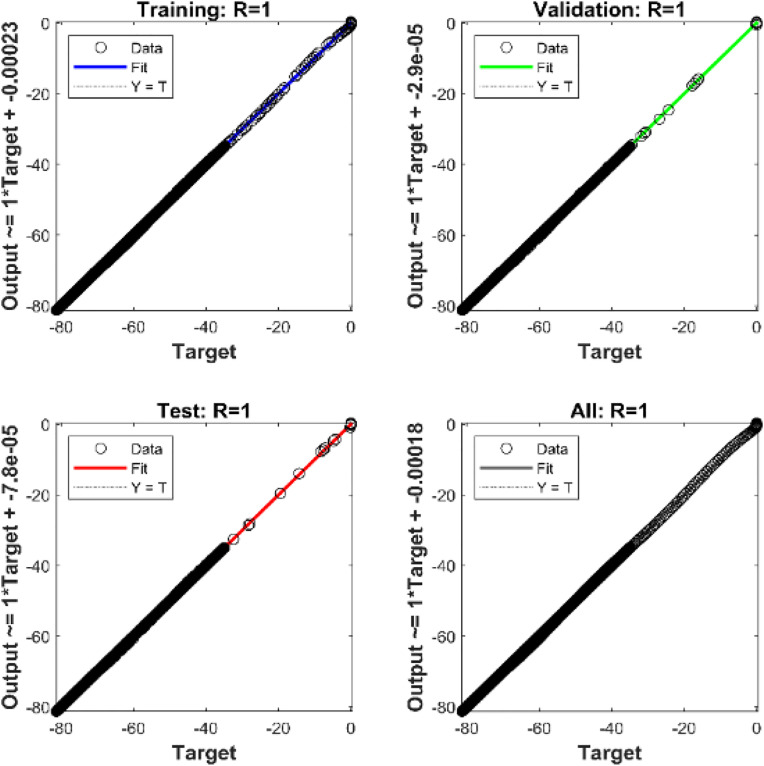
Correlation coefficient (R).

To evaluate the effectiveness and performance of the suggested ANN-BSC control strategy, the entire system was simulated using the MATLAB/Simulink software environment. Table 2 displays the parameter values of the wind power conversion system used during the simulation phase. A harmonic variable wind model was adopted for 5 seconds, as shown in Fig 6. The proposed ANN-based MPPT controller performs well, as demonstrated by the minimal value ($\text{1},\text{066}.{\text{10}}^{-\text{3}}$) of MSE shown in Fig 7. In addition, analyzing the regression plotted in Fig 8, and considering the correlation coefficient value (R = 1) reveals a high level of agreement between the output and goal values.

**Table 2:** System parameters.

Wind Turbine Parameters	PMSG Parameters	FOC-PI Parameters	BSC Parameters
$\rho =1.225kg/m^{3}$	$\varphi_{r}=\text{0}.\text{125}Wb$	$k_{pd}=\text{0}.\text{42}$	$\gamma_{\text{1}}=\text{1000}$
R=2m	$R_{s}=\text{0}.\text{6}\Omega$	$k_{id}=\text{180}$	$\gamma_{\text{2}}=\text{60000}$
$C_{p,max}=0,48$	$L_{sd}=L_{sq}=\text{1}.\text{4mH}$	$k_{pq}=\text{0}.\text{42}$	$\gamma_{\text{3}}=\text{60000}$
$\lambda_{opt}=8,1$	p=8	$k_{iq}=\text{180}$	
$\beta =0^{\circ }$			
$J=0.02kg.m^{2}$			
$f_{c}=1.5e-3N.m.s/rad$			

**Fig 9:**
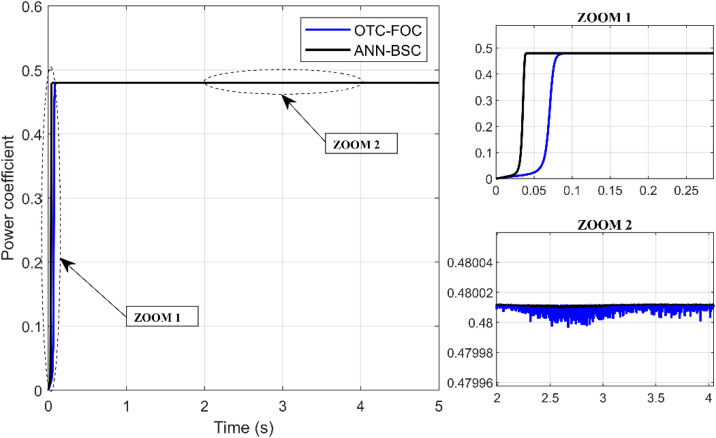
Evolution.

**Fig 10:**
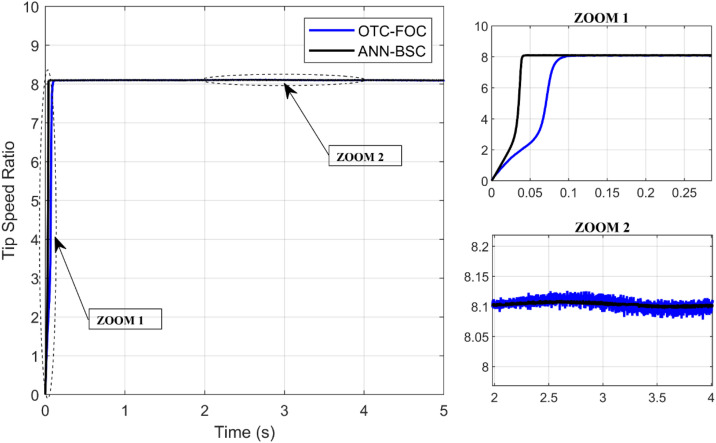
Tip speed ratio.

**Fig 11:**
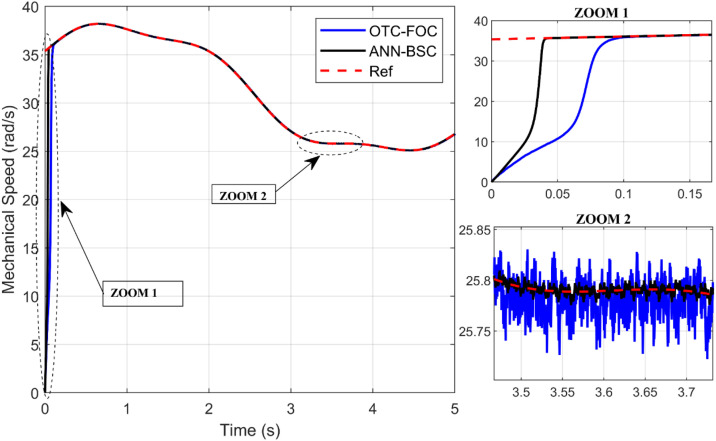
Mechanical speed.

**Fig 12:**
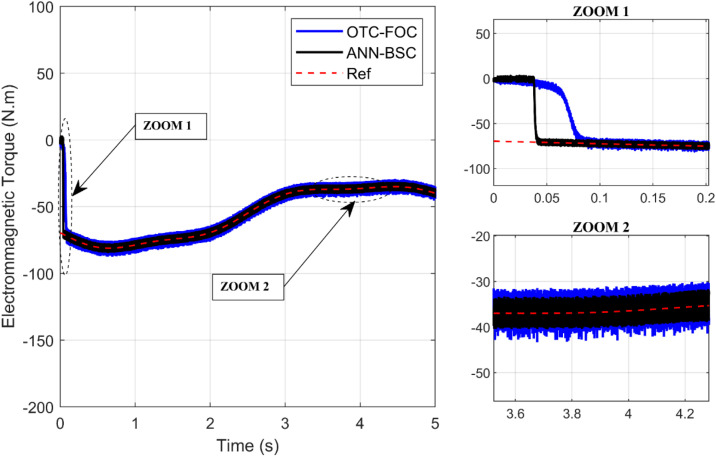
Electromagnetic torque.


On the one hand, Fig 9 and Fig 10 show that although the wind speed varies, both control strategies allow the system to achieve and maintain the optimal values of the $C_{p}$ and the TSR $\left(C_{p,max}=\text{0},\text{48};\lambda_{opt}=\text{8},\text{1}\right)$, respectively, which means that the maximum amount of energy is extracted from the wind. However, the proposed technique ANN-BSC guarantees less fluctuations compared to strategy based on OTC and conventional FOC as illustrated in the Zoom parts. Moreover, despite variations in wind speed, the generator’s mechanical speed follows its reference for both control strategies. But more precisely when ANN-BSC control is applied as shown in Fig 11, it ensures that the system operates at its optimum speed. Nevertheless, Fig 12 clearly illustrates that the electromagnetic torque follows its reference, but shows less ripples for ANN-BSC control versus OTC-FOC. In order to analyze and compare the results regarding the ripple in the electromagnetic torque of PMSG, a time interval of 0.3 seconds has been randomly selected between instants 3.5s and 3.8s. The ripple percentage $\%T_{em}^{ripple}$ was calculated by applying the following formula to each control strategy separately, and the results are shown in Table 3.

**Table 3:** Electromagnetic torque ripple comparison.

Control strategy	OTC-FOC strategy	Proposed strategy: ANN-BSC
$T_{em}^{Max}$ Maximum torque	−31,42 N.m	−33,32 N.m
$T_{em}^{Min}$ Minimum torque	−43,60 N.m	−40,50 N.m
$T_{em}^{Avg}$ Average torque	−36,96 N.m	−36,95 N.m
$\%T_{em}^{ripple}$	32,95%	19,43%

$$\%T_{em}^{ripple}=100\times \left|\frac{T_{em}^{Max}-T_{em}^{Min}}{T_{em}^{Avg}}\right|$$ (38)

**Fig 13:**
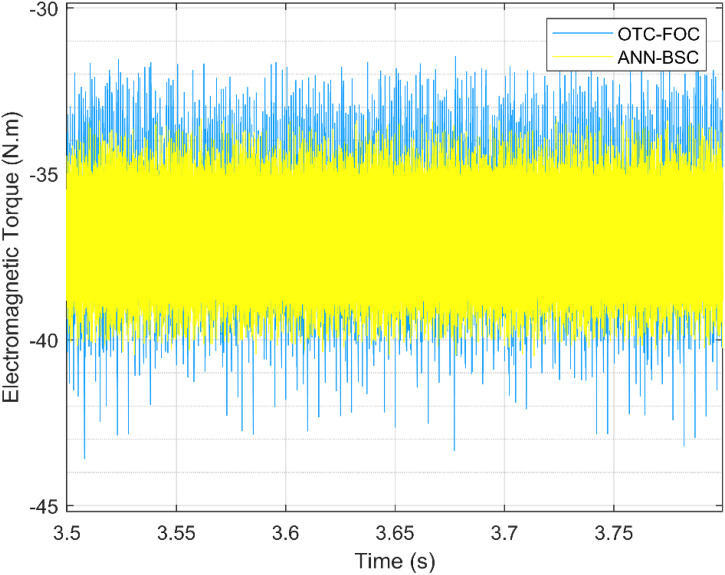
Electromagnetic torque ripple.

Compared to the results of the conventional method (OTC-FOC), the proposed hybrid method shows less ripples in the electromagnetic torque, as the percentage of ripples is significantly reduced from 32,95% to 19,43% while keeping the average torque approximately constant at 36,96 N.m during the same time scale as displayed in Fig 13, which reflects the high effectiveness of the proposed technique.

**Fig 14:**
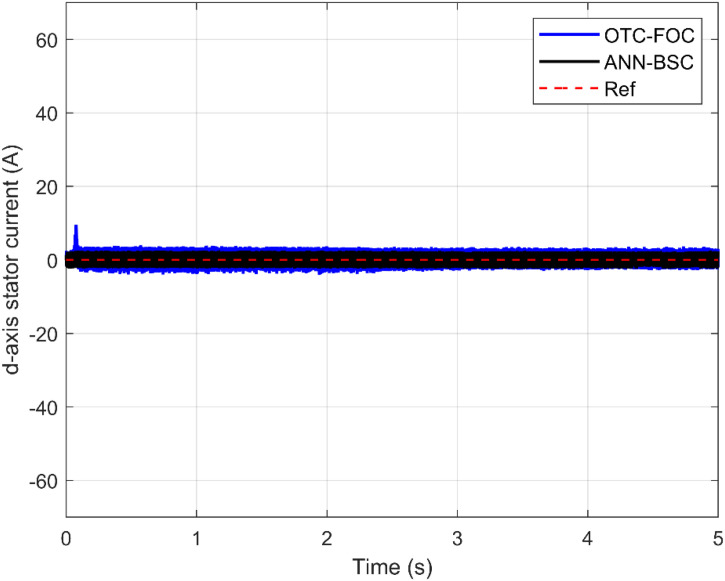
d-axis current.

**Fig 15:**
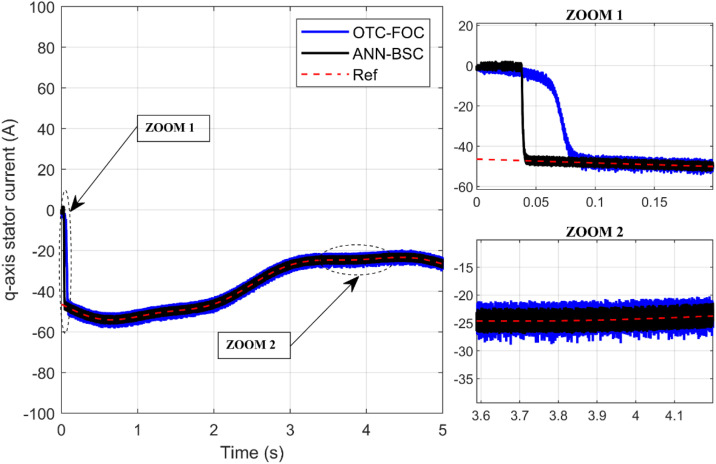
q-axis current.

**Fig 16:**
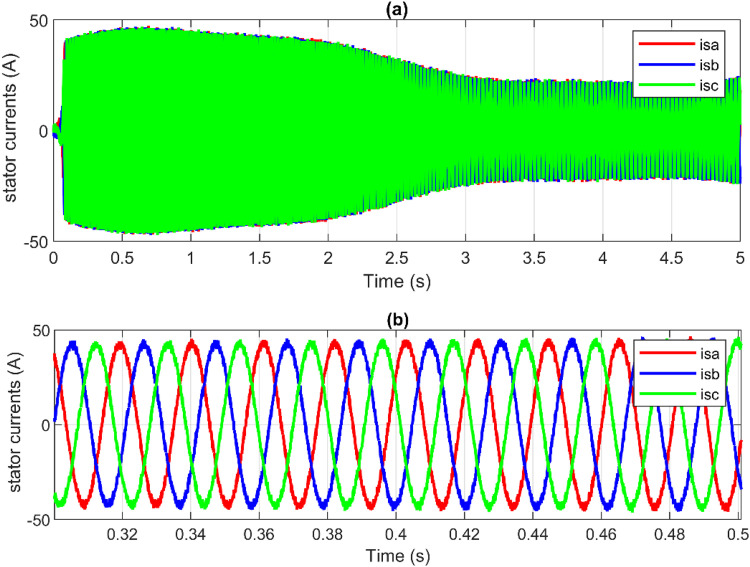
OTC-FOC outcomes: (a) PMSG currents, (b) Zoom-in illustration.

**Fig 17:**
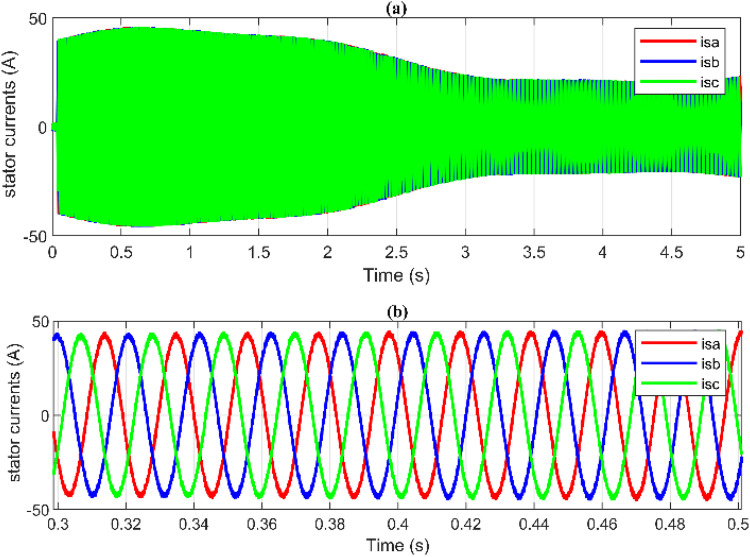
ANN-BSC outcomes: (a) PMSG currents, (b) Zoom-in illustration.

Additionally, Fig 14 and Fig 15 show the response of the PMSG current components and demonstrate that both component$i_{sd}$ and $i_{sq}$ are tracking their respective reference values $i_{sd}^{*}$ and $i_{sq}^{*}$. The electromagnetic torque is directly proportional to the stator currents, which also causes ripples in the stator currents. Additionally, among the benefits of the proposed hybrid control strategy is minimizing these current ripples as shown in Fig 16 and Fig 17. Furthermore, high-quality sinusoidal waveforms of the phase PMSG currents are achieved based on ANN-BSC versus OTC-FOC.

**Fig 18:**
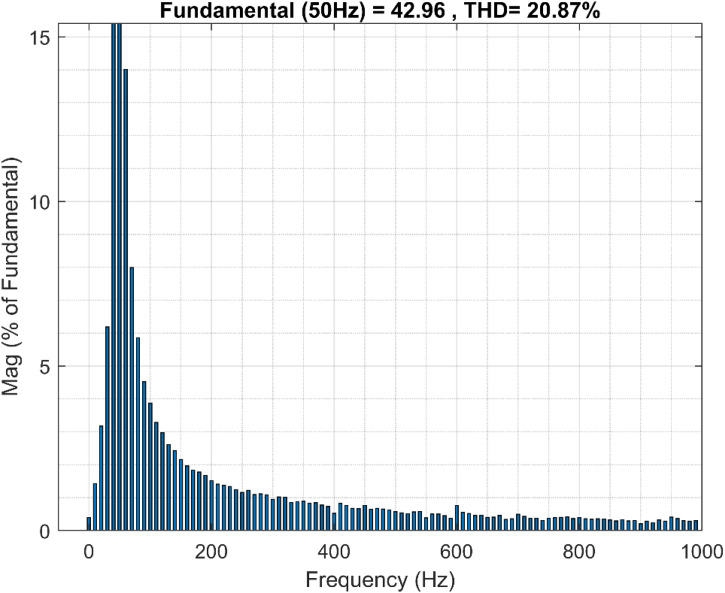
THD for the stator current i_sb_ (OTC-FOC).

**Fig 19:**
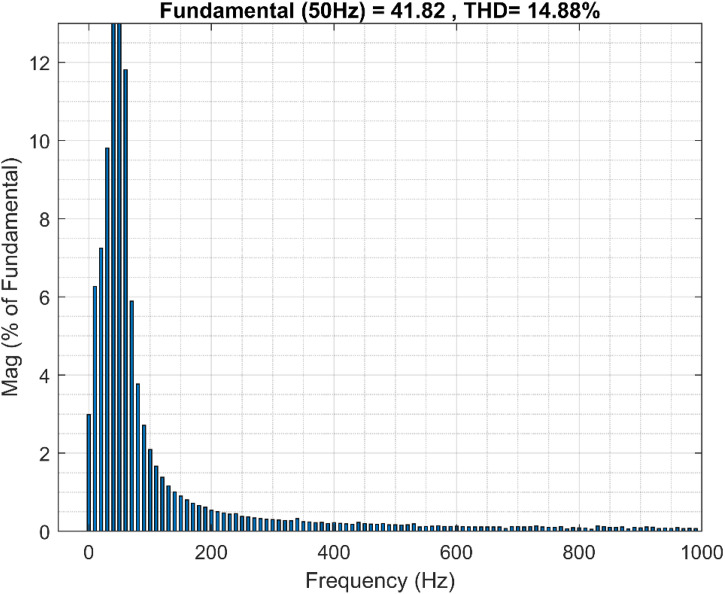
THD for the stator current i_sb_ (ANN-BSC).

Moreover, the results of the fast Fourier transform (FFT) studies of the PMSG stator currents reveal the effectiveness of the ANN-BSC control strategy compared to the OTC-FOC control strategy, as proved in the spectral analysis of the total harmonic distortion (THD) of the PMSG currents. The proposed control strategy significantly reduced the THD values, 14.88% compared to 20.87% for OTC-FOC as illustrated in Fig 18 and Fig 19.

The performance metrics in Table 4 reveal a clear picture of the superiority of the proposed control strategy over the OTC-FOC strategy. The ANN-BSC technique reduces rise time, transient time, and settling time from 16.9 ms to 7.1 ms, 81.0 ms to 38.2 ms, and 81.0 ms to 38.2 ms, respectively. The undershoot value is 0 for both control strategies. In contrast, the overshoot is about 0.002% and 6.8835e-05% for OTC-FOC and ANN-BSC, respectively. Another performance parameter reveals that the ANN-BSC presents excellent setpoint tracking over the OTC-FOC. These results show the high performance of the proposed ANN-BSC control compared to the OTC-FOC, which makes it a suitable solution for WECS under variable wind conditions.

**Table 4:** Performance comparison between the OTC-FOC and ANN-BSC.

Control strategy	OTC-FOC strategy	Proposed strategy: ANN-BSC
Rise time (ms)	16.9	7.1
Transient time (ms)	81.0	38.2
Settling time (ms)	81.0	38.2
Overshoot (%)	0.0020	6.88e-05
Undershoot (%)	0	0
Set-point tracking	Good	Excellent

On the other hand, the control algorithm’s complexity is a crucial factor in determining memory requirements. The proposed ANN-BSC is a hybrid nonlinear and complex control strategy that requires significant memory and processing resources to execute its advanced algorithms. In addition, this control strategy requires a deep understanding of control theory, which translates into higher implementation costs. In contrast, conventional FOC control is a simple linear control strategy that needs less expensive devices and software. Table 5 summarizes the main features of both control methods ANN-BSC and OTC-FOC.

**Table 5:** Summary comparison of proposed ANN-BSC control strategy versus OTC-FOC strategy.

Control strategy	OTC-FOC strategy	Proposed strategy: ANN-BSC
Control System	Linear	Nonlinear
Implementation Complexity	Simple	Complex
Robustness	Low	Very high
Torque/Current Ripple	Moderate	Low
Efficiency	Moderate	High
Performance	Low	High
Memory requirement	No	Yes
Anemometer requirement	No	Yes
Cost	Moderate	High
PMSG Current THD	20.87%	14.88%

## 5. Conclusion

This present paper proposes and designs a hybrid nonlinear robust control strategy known as combined BSC with ANN technique for direct drive WECS based on PMSG. Compared to FOC, despite its high cost, memory requirement, and complexity, the proposed control strategy offers superior robustness and performance, making it more suitable for nonlinear and fluctuating conditions in WECS. The proposed control strategy optimized the transition regime of the studied system. It also reduced the apparent ripples in the electromagnetic torque and stator currents of PMSG. Hence, it ensures the optimal operation of WECS to extract the maximum available power from wind power.

From the perspective of this research, future work will focus on the design of adaptive backstepping control for the grid-side converter of the PMSG-based WECS to ensure the quality of the injected electric power and the stability of the electric grid at the same time. Moreover, a real-time implementation of the work will be attempted using the dSPACE DS1104 prototyping platform to verified the simulation results.

On the other hand, future research directions focus on developing hybrid technologies in more challenging and dynamic WECS environments, including various technologies used in multi-rotor wind turbines, wind energy-based electricity storage, and large-scale harnessing of wind energy for green hydrogen production.

## References

[1] Mebkhouta T, Golea A, Boumaraf R, Benchouia TM, Karboua D, Bajaj M, et al. Sensorless finite set predictive current control with MRAS estimation for optimized performance of standalone DFIG in wind energy systems. Results Eng. 2024;24:103622. doi: 10.1016/j.rineng.2024.103622

[2] Boadu S, Otoo E. A comprehensive review on wind energy in Africa: challenges, benefits and recommendations. Renew Sustain Energy Rev. 2024;191:114035. doi: 10.1016/j.rser.2023.114035

[3] GWEC. Global wind report 2024. 2024. www.gwec.net

[4] GWEC. “WIND IN AFRICA,” no. 2023.

[5] Dani A, Mekrini Z, El Mrabet M, Boulaala M. A review of different structures generators and control strategies applied to the wind turbine. In: Lecture notes in networks and systems. Springer Nature Switzerland; 2023. 190–9. doi: 10.1007/978-3-031-35245-4_18

[6] Mousa HHH, Youssef A-R, Mohamed EEM. Hybrid and adaptive sectors P&O MPPT algorithm based wind generation system. Renewable Energy. 2020;145:1412–29. doi: 10.1016/j.renene.2019.06.078

[7] Meda S, Muni BP, Reddy KR. Performance analysis of OTC and improved PSO MPPT techniques for DFIG-based wind energy conversion systems. SSRG-IJEEE. 2023;10(8):215–23. doi: 10.14445/23488379/ijeee-v10i8p121

[8] Sathish Babu P, Sundarabalan CK, Balasundar C, Santhana Krishnan T. Fuzzy logic based optimal tip speed ratio MPPT controller for grid connected WECS. Materials Today: Proceedings. 2021;45:2544–50. doi: 10.1016/j.matpr.2020.11.259

[9] Mossa MA, Mohamed RA, Al-Sumaiti AS. Performance enhancement of a grid connected wind turbine-based PMSG using effective predictive control algorithm. IEEE Access. 2025;13:64160–85. doi: 10.1109/access.2025.3557194

[10] Mossa MA, Gam O, Bianchi N, Quynh NV. Enhanced control and power management for a renewable energy-based water pumping system. IEEE Access. 2022;10:36028–56. doi: 10.1109/access.2022.3163530

[11] Ding M, Tao Z, Hu B, Ye M, Ou Y, Yokoyama R. A fuzzy control and neural network based rotor speed controller for maximum power point tracking in permanent magnet synchronous wind power generation system. Global Energy Intercon. 2023;6(5):554–66. doi: 10.1016/j.gloei.2023.10.004

[12] Mossa MA, Echeikh H, Quynh NV, Bianchi N. Performance dynamics improvement of a hybrid wind/fuel cell/battery system for standalone operation. IET Renewable Power Gen. 2022;17(2):349–75. doi: 10.1049/rpg2.12603

[13] Wind energy conversion technologies and control strategies: a review. Int J Renew Energy Res. 2024;(v11i2). doi: 10.20508/ijrer.v14i1.14296.g8869

[14] Karboua D, Belgacem T, Khan ZH, Kellal C. Robust performance comparison of PMSM for flight control applications in more electric aircraft. PLoS One. 2023;18(7):e0283541. doi: 10.1371/journal.pone.0283541 37418360 PMC10328255

[15] Li Z, Cai J, Wang L, Liu X, Wang L, Liu L, et al. A comparative study and validation of super-twisting sliding mode control for PMSG-based wind power systems. Control Eng Practice. 2025;164:106488. doi: 10.1016/j.conengprac.2025.106488

[16] Eial Awwad A. Dynamic performance enhancement of a direct-driven PMSG-based wind turbine using a 12-sectors DTC. WEVJ. 2022;13(7):123. doi: 10.3390/wevj13070123

[17] Sahri Y, Tamalouzt S, Lalouni Belaid S, Bajaj M, Belkhier Y, Singh AR, et al. Effectiveness analysis of twelve sectors of DTC based on a newly modified switching table implemented on a wind turbine DFIG system under variable wind velocity. Ain Shams Eng J. 2023;14(11):102221. doi: 10.1016/j.asej.2023.102221

[18] Chojaa H, Derouich A, Zamzoum O, Watil A, Taoussi M, Abdelaziz AY, et al. Robust control of DFIG-based wecs integrating an energy storage system with intelligent MPPT under a real wind profile. IEEE Access. 2023;11:90065–83. doi: 10.1109/access.2023.3306722

[19] Wiam A, Ali H. Direct torque control-based power factor control of a DFIG. Energy Procedia. 2019;162:296–305. doi: 10.1016/j.egypro.2019.04.031

[20] Sayeh KF, Tamalouzt S, Sahri Y, Lalouni Belaid S, Bekhiti A. Artificial intelligence-based direct power control for power quality improvement in a WT-DFIG system via neural networks: prediction and classification techniques. J Franklin Instit. 2025;362(1):107401. doi: 10.1016/j.jfranklin.2024.107401

[21] El Fadili Y, Berrada Y, Boumhidi I. Improved sliding mode control law for wind power systems. Int J Dynam Control. 2024;12(9):3354–65. doi: 10.1007/s40435-024-01431-6

[22] Mensou S, Essadki A, Nasser T, Bououlid Idrissi B. A direct power control of a DFIG based-WECS during symmetrical voltage dips. Prot Control Mod Power Syst. 2020;5(1). doi: 10.1186/s41601-019-0148-y

[23] Tidjani N, Guessoum A. Augmented robust T-S fuzzy control based PMSG wind turbine improved with H∞ performance. IJPEDS. 2021;12(1):585. doi: 10.11591/ijpeds.v12.i1.pp585-596

[24] Kahla S, Bechouat M, Amieur T, Sedraoui M, Babes B, Hamouda N. Maximum power extraction framework using robust fractional-order feedback linearization control and GM-CPSO for PMSG-based WECS. Wind Engineering. 2020;45(4):1040–54. doi: 10.1177/0309524x20948263

[25] Ranjineh Khojasteh A, Toshani H. Design nonlinear feedback strategy using H2/H∞ control and neural network based estimator for variable speed wind turbine. Int J Dynam Control. 2021;10(2):447–61. doi: 10.1007/s40435-021-00813-4

[26] Islam MS, Bushra IJ, Roy TK, Oo AMT. Virtual capacitor‐based robust composite controller for stability enhancement in DC microgrids with wind, PV and battery integration. IET Generation Trans Dist. 2025;19(1). doi: 10.1049/gtd2.70125

[27] Faraji Nayeh R, Moradi H, Vossoughi G. Multivariable robust control of a horizontal wind turbine under various operating modes and uncertainties: a comparison on sliding mode and H∞ control. Inter J Electr Power Energy Syst. 2020;115:105474. doi: 10.1016/j.ijepes.2019.105474

[28] Debouza M, Al-Durra A. Design of H-infinity controller for doubly fed induction generator based wind turbine. In: 2019 IEEE 28th International Symposium on Industrial Electronics (ISIE), 2019. 491–6. doi: 10.1109/isie.2019.8781309

[29] Utkin V. Variable structure systems with sliding modes. IEEE Trans Automat Contr. 1977;22(2):212–22. doi: 10.1109/tac.1977.1101446

[30] El Fadili Y, Boumhidi I. Enhanced nonlinear sliding mode control technique for wind power generation systems application: Theoretical design and comparative study. e-Prime - Adv Electrical Eng Electron Energy. 2025;11:100937. doi: 10.1016/j.prime.2025.100937

[31] Karboua D, Toual B, Kouzou A, Douara BO, Mebkhouta T, Bendenidina AN. High-order supper-twisting based terminal sliding mode control applied on three phases permanent synchronous machine. Period Polytech Elec Eng Comp Sci. 2023;67(1):40–50. doi: 10.3311/ppee.21026

[32] Mahfoud S, El Ouanjli N, Derouich A, El Idrissi A, Chetouani E, Loulijat A, et al. An advanced direct torque control for doubly fed induction motor using evolutionary computational techniques. Sci Rep. 2025;15(1):22719. doi: 10.1038/s41598-025-08287-6 40596623 PMC12214836

[33] Kanellakopoulos I, Kokotovic PV, Morse AS. Systematic design of adaptive controllers for feedback linearizable systems. In: 1991 American Control Conference, 1991. doi: 10.23919/acc.1991.4791451

[34] Mossa MA, Echeikh H, Quynh NV. A novel sensorless predictive voltage control for an induction motor drive based on a back-stepping observer-experimental validation. IEEE Access. 2021;9:11921–42. doi: 10.1109/access.2021.3051436

[35] Liu Y. Adaptive dynamic surface asymptotic tracking for a class of uncertain nonlinear systems. Intl J Robust Nonlinear. 2017;28(4):1233–45. doi: 10.1002/rnc.3947

[36] Echiheb F, Ihedrane Y, Bossoufi B, Bouderbala M, Motahhir S, Masud M, et al. Robust sliding-backstepping mode control of a wind system based on the DFIG generator. Sci Rep. 2022;12(1):11782. doi: 10.1038/s41598-022-15960-7 35821271 PMC9276821

[37] Chahboun M, Abouyaakoub M, Ali AA, El Mrabet A, Hihi H, Ouabi H, et al. Backstepping approach for the control of the double-fed asynchronous generator in a wind power system. IJEECS. 2025;37(1):78. doi: 10.11591/ijeecs.v37.i1.pp78-89

[38] Benzaoui K, Bouguerra A, Zeghlache S, Elsanabary A, Mekhilef S, Bendib A, et al. SABO optimization algorithm-based backstepping controller for DSIG within a wind turbine system. Electr Eng. 2024;107(5):5939–55. doi: 10.1007/s00202-024-02839-1

[39] Bossoufi B, Karim M, Taoussi M, Aroussi HA, Bouderbala M, Deblecker O, et al. Rooted tree optimization for the backstepping power control of a doubly fed induction generator wind turbine: dSPACE implementation. IEEE Access. 2021;9:26512–22. doi: 10.1109/access.2021.3057123

[40] Munteanu I, Cutululis NA, Bratcu AI, Ceangă E. Optimization of variable speed wind power systems based on a LQG approach. Control Eng Practice. 2005;13(7):903–12. doi: 10.1016/j.conengprac.2004.10.013

[41] Mossa MA, Ouanjli NE, Gam O, Kamel OM. Performance improvement of a hybrid energy system feeding an isolated load. In: 2022 23rd International Middle East Power Systems Conference (MEPCON), 2022. 1–8. doi: 10.1109/mepcon55441.2022.10021715

[42] Jiang P, Zhang T, Geng J, Wang P, Fu L. An MPPT strategy for wind turbines combining feedback linearization and model predictive control. Energies. 2023;16(10):4244. doi: 10.3390/en16104244

[43] Mebkhouta T, Golea A, Boumaraf R, Benchouia TM, Karboua D. A high robust optimal nonlinear control with MPPT Speed for Wind Energy Conversion System (WECS) based on Doubly Fed Induction Generator (DFIG). Period Polytech Elec Eng Comp Sci. 2023;68(1):1–11. doi: 10.3311/ppee.22595

[44] Shengquan L, Juan L, Yongwei T, Yanqiu S, Wei C. Model-based model predictive control for a direct-driven permanent magnet synchronous generator with internal and external disturbances. Transac Instit Measure Control. 2019;42(3):586–97. doi: 10.1177/0142331219878574

[45] Belabbes A. Advanced control of PMSG-based Wind energy conversion system using model predictive and sliding mode control. Electrotech Rev. 2024;1(2):12–8. doi: 10.15199/48.2024.02.02

[46] Chaturvedi P, Palwalia DK. Passivity-based fuzzy logic approach for optimal power extraction from PMSG-wind energy conversion. IJPEDS. 2024;15(3):1826. doi: 10.11591/ijpeds.v15.i3.pp1826-1837

[47] Majout B, Bossoufi B, Karim M, Skruch P, Mobayen S, El Mourabit Y, et al. Artificial neural network-based direct power control to enhance the performance of a PMSG-wind energy conversion system under real wind speed and parameter uncertainties: an experimental validation. Energy Reports. 2024;11:4356–78. doi: 10.1016/j.egyr.2024.03.039

[48] Tareq WZT. (Artificial) neural networks. In: Decision-making models. Elsevier; 2024. 329–37. doi: 10.1016/b978-0-443-16147-6.00035-9

[49] Ouhssain S, Chojaa H, Aljarhizi Y, Al Ibrahmi E, Maarif A, A. Mossa M. Enhancing the performance of a wind turbine based DFIG generation system using an effective ANFIS control technique. IJRCS. 2024;4(4):1617–40. doi: 10.31763/ijrcs.v4i4.1451

[50] Aounallah T, Essounbouli N, Hamzaoui A, Bouchafaa F. Algorithm on fuzzy adaptive backstepping control of fractional order for doubly‐fed induction generators. IET Renewable Power Gen. 2018;12(8):962–7. doi: 10.1049/iet-rpg.2017.0342

[51] Sakouchi A, Djahbar A, Bounadja E, Benbouhenni H, Iqbal A, Moualdia A, et al. Enhanced control of grid-connected multi-machine wind power generation systems using fuzzy backstepping approaches. Energy Reports. 2024;12:4208–31. doi: 10.1016/j.egyr.2024.09.077

[52] Veisi A, Delavari H. Adaptive fractional backstepping intelligent controller for maximum power extraction of a wind turbine system. J Renew Sustain Energy. 2023;15(6). doi: 10.1063/5.0161571

[53] Jaramillo-Lopez F, Kenne G, Lamnabhi-Lagarrigue F. A novel online training neural network-based algorithm for wind speed estimation and adaptive control of PMSG wind turbine system for maximum power extraction. Renewable Energy. 2016;86:38–48. doi: 10.1016/j.renene.2015.07.071

[54] Labdai S, Hemici B, Nezli L, Bounar N, Boulkroune A, Chrifi-Alaoui L. Robust Control based on Backstepping and adaptive neural network for the DFIG based WECS. In: 2019 International Conference on Control, Automation and Diagnosis (ICCAD), 2019. 1–6. doi: 10.1109/iccad46983.2019.9037898

[55] Beniysa M, Janati El Idrissi AE, Bouajaj A, Réda Britel M, Ariwa E. Neural network adaptive backstepping control via uncertainty compensation for PMSG-based variable-speed wind turbine: Controller design and stability analysis. Wind Engineering. 2021;46(2):439–58. doi: 10.1177/0309524x211031269

[56] Mansouri A, El Magri A, El Myasse I, Lajouad R, Elaadouli N. Backstepping nonlinear control of a five-phase PMSG aerogenerator linked to a Vienna rectifier. IJEECS. 2023;32(2):734. doi: 10.11591/ijeecs.v32.i2.pp734-741

[57] Hattab W, Benakcha A, Tabet S, Slimani A. Systems using PMSG with backstepping and ANN-based MPPT. J Eng Technol Ind Appl. 2025;1152:22–33. doi: 10.5935/jetia.v11i52.1477

[58] Fannakh M, Larbi Elhafyani M, Zouggar S, Zahboune H. Overall fuzzy logic control strategy of direct driven PMSG wind turbine connected to grid. IJECE. 2021;11(6):5515. doi: 10.11591/ijece.v11i6.pp5515-5529

[59] Rekioua D, Mezzai N, Mokrani Z, Oubelaid A, Kakouche K, Logerais PO, et al. Effective optimal control of a wind turbine system with hybrid energy storage and hybrid MPPT approach. Sci Rep. 2024;14(1):30013. doi: 10.1038/s41598-024-78847-9 39622862 PMC11612302

[60] Castillo OC, Andrade VR, Rivas JJR, González RO. Comparison of power coefficients in wind turbines considering the tip speed ratio and blade pitch angle. Energies. 2023;16(6):2774. doi: 10.3390/en16062774

[61] Mohanraj D, Gopalakrishnan J, Chokkalingam B, Mihet-Popa L. Critical aspects of electric motor drive controllers and mitigation of torque ripple—review. IEEE Access. 2022;10:73635–74. doi: 10.1109/access.2022.3187515

[62] Pan L, Shao C. Wind energy conversion systems analysis of PMSG on offshore wind turbine using improved SMC and extended state observer. Renewable Energy. 2020;161:149–61. doi: 10.1016/j.renene.2020.06.057

[63] Ali SW, Verma AK, Terriche Y, Sadiq M, Su C-L, Lee C-H, et al. Finite-control-set model predictive control for low-voltage-ride-through enhancement of PMSG based wind energy grid connection systems. Mathematics. 2022;10(22):4266. doi: 10.3390/math10224266

[64] Mousa HHH, Youssef A-R, Hamdan I, Ahamed M, Mohamed EEM. Performance assessment of robust P&O algorithm using optimal hypothetical position of generator speed. IEEE Access. 2021;9:30469–85. doi: 10.1109/access.2021.3059884

[65] Chojaa H, Derouich A, Taoussi M, Chehaidia S, Zamzoum O, Mosaad M, et al. Nonlinear control strategies for enhancing the performance of DFIG-based WECS under a real wind profile. Energies. 2022;15(18):6650. doi: 10.3390/en15186650

[66] Hamid C, Derouich A, Taoussi M, Zamzoum O, Hanafi A. An improved performance variable speed wind turbine driving a doubly fed induction generator using sliding mode strategy. In: 2020 IEEE 2nd International Conference on Electronics, Control, Optimization and Computer Science (ICECOCS), 2020. 1–8. doi: 10.1109/icecocs50124.2020.9314629

[67] Zamzoum O, Derouich A, Motahhir S, El Mourabit Y, El Ghzizal A. Performance analysis of a robust adaptive fuzzy logic controller for wind turbine power limitation. J Clean Prod. 2020;265:121659. doi: 10.1016/j.jclepro.2020.121659

[68] Dani A, Mekrini Z, El Mrabet M, Boulaala M. Maximum power point tracking and field-oriented control strategies applied to PMSG-based WECS. In: Lecture notes in networks and systems. Springer Nature Switzerland; 2024. 506–16. doi: 10.1007/978-3-031-68650-4_48

[69] Ramesh P, Umavathi M, Bharatiraja C, Ramanathan G, Athikkal S. Development of a PMSM motor field-oriented control algorithm for electrical vehicles. Materials Today: Proceed. 2022;65:176–87. doi: 10.1016/j.matpr.2022.06.080

[70] Li X, He J, Wen C, Liu X-K. Backstepping-based adaptive control of a class of uncertain incommensurate fractional-order nonlinear systems with external disturbance. IEEE Trans Ind Electron. 2022;69(4):4087–95. doi: 10.1109/tie.2021.3070513

[71] Liu X-D, Li K, Zhang C-H. Improved backstepping control with nonlinear disturbance observer for the speed control of permanent magnet synchronous motor. J Electr Eng Technol. 2019;14(1):275–85. doi: 10.1007/s42835-018-00021-9

[72] Wang H, Watson JD, Watson NR. A Lyapunov-based nonlinear direct power control for grid-side converters interfacing renewable energy in weak grids. Electric Power Syst Res. 2023;221:109408. doi: 10.1016/j.epsr.2023.109408

[73] Mekrini Z, Bri S. High –performance using neural networks in direct torque control for asynchronous machine. Int J Electr Comput Eng. 2018;8(2):1010. doi: 10.11591/ijece.v8i2.pp1010-1017

[74] Chojaa H, Derouich A, Chehaidia SE, Zamzoum O, Taoussi M, Elouatouat H. Integral sliding mode control for DFIG based WECS with MPPT based on artificial neural network under a real wind profile. Energy Rep. 2021;7:4809–24. doi: 10.1016/j.egyr.2021.07.066

